# Analysis of the dentin-pulp complex in teeth submitted to orthodontic movement in rats

**DOI:** 10.1590/S1678-77572009000700007

**Published:** 2009

**Authors:** Camila da Siveira MASSARO, Renata Bianco CONSOLARO, Milton SANTAMARIA, Maria Fernanda Martins-Ortiz CONSOLARO, Alberto CONSOLARO

**Affiliations:** 1 Undergraduate student, Bauru School of Dentistry, University of São Paulo, SP, Brazil; 2 DDS, MSc, PhD student of Oral Pathology, Bauru School of Dentistry, University of São Paulo, SP, Brazil; 3 DDS, MSc, PhD, Orthodontics Practice in Ribeirão Preto, SP, Brazil; 4 DDS, MSc, PhD, Professor of Orthodontics and Oral Biology, University of Sacred Heart, Bauru, SP, Brazil; 5 Professor of Oral Pathology, Department of Stomatology, Bauru School of Dentistry, University of São Paulo, SP, Brazil

**Keywords:** Dental pulp, Rat, Tooth movement, Root resorption

## Abstract

In order to microscopically analyze the pulpal effects of orthodontic movement, 49 maxillary first molars of rats were submitted to orthodontic appliance composed of a closed coil spring anchored to the maxillary incisors, placed for the achievement of mesial movement. Material and Methods: Ten animals were used as the control group and were not submitted to orthodontic force; the other animals were divided into groups according to the study period of tooth movement, namely 1, 2, 3, 4, 5, 6 and 7 days. The investigation of pulp and periodontal changes included hyalinization, fibrosis, reactive dentin and vascular congestion. Statistical evaluation was performed between control and experimental groups and between periods of observation using non-parametric chi-square, Kruskal-Wallis and Dunn tests. Results: There was no statistically significant difference concerning pulpal changes between control and experimental groups nor between periods of observation. The control group, at 3 and 5 days, revealed greater hyalinization of the periodontal ligament (p<0.05), whereas root resorption was significantly greater at 5 and 7 days (p<0.05). Conclusion: No morphological change from the effect of induced tooth movement could be found in the dentin-pulp complex. In addition, no inflammatory or pulp degeneration, detectable in optical microscopy, was found in experimental groups.

## INTRODUCTION

The pulp and periodontal tissues are richly cellularized, and their metabolic rates are adapted to their functional needs. The structural and functional normality of these tissues seem to be influenced by local and systemic factors^7^. Clinical detection of periodontal and pulp changes induced by local and systemic factors probably depend on the type, duration and intensity of the stimulus applied.

Early studies have suggested that vascular changes promoted by orthodontic movement might cause pulp necrosis. Oppenheim^20^^–^^22^, in 1936, 1937 and 1942, affirmed that teeth submitted to orthodontic movement presented occasional atresia of the pulp and root canal, observed by radiographic examination. Microscopically, there was an increase in collagen fibers and inflammatory cells and a reduction in blood vessels, indicating probable pulp degeneration.

Strang^29^ (1943) suggested that abrupt forces on teeth, especially intrusion, might damage the blood vessels in the pulp, causing blood vessel congestion and pulp necrosis. Butcher and Taylor^3^^,^^4^, in 1951 and 1952, applied retraction forces to incisors of monkeys and reported the occurrence of pulp necrosis.

In their study, Mjör and Stenvik^19^ (1969) applied intrusion forces to human teeth and did not observe any significant differences in the pulp tissue of experimental and control groups; the observation of vacuolization in the pulp tissue was considered to be an artifact. Anstendig and Kronman1 conducted an investigation on dogs and also observed vacuolization of the odontoblast layer as the main pulp changed after orthodontic movement.

In the 1980s, Hamersky, et al.^12^ and Unsterseher, et al.^31^ (1987) investigated biochemically the effect of orthodontic forces on the respiratory metabolism of the pulp of human teeth, with the aid of radioactive carbon dioxide. The authors concluded that pulp respiration is reduced in orthodontically moved teeth.

Studies using laser doppler flowmetry conducted by McDonald and Pitt Ford^18^ (1994), Ikawa, et al.^14^ (2001) and Sano, et al.^26^ (2002) revealed that application of forces on human teeth reduced the initial blood flow to the pulp, in disagreement with the findings of Barwick and Ransay^2^ (1996), which did not reveal any changes.

Encouraged by the initial flowmetry studies, Derriger, et al.^9^^,^^10^ (1996, 1998) analyzed the secretion of angiogenic factors on human teeth and observed that these factors were increased in orthodontically moved teeth. Santamaria, et al.^28^ (2006), detected a slight increase in the volume of blood vessels only during the first 6 hours of induced tooth movement in molars of rats. Ramazanzadeh, et al.^25^ (2009), described similar results in human teeth submitted to intrusive and extrusive forces.

The related literature presents variable models and criteria for analysis, as well as results and conclusions. Thus, the following doubts remain: 1. May induced tooth movement promote early pulp aging, even if only microscopically observable? 2. May it promote pulpitis, pulp necrosis or any other change? These questions seem to remain without answers and were the initial premises of this study.

## MATERIAL AND METHODS

**Figure 1:**
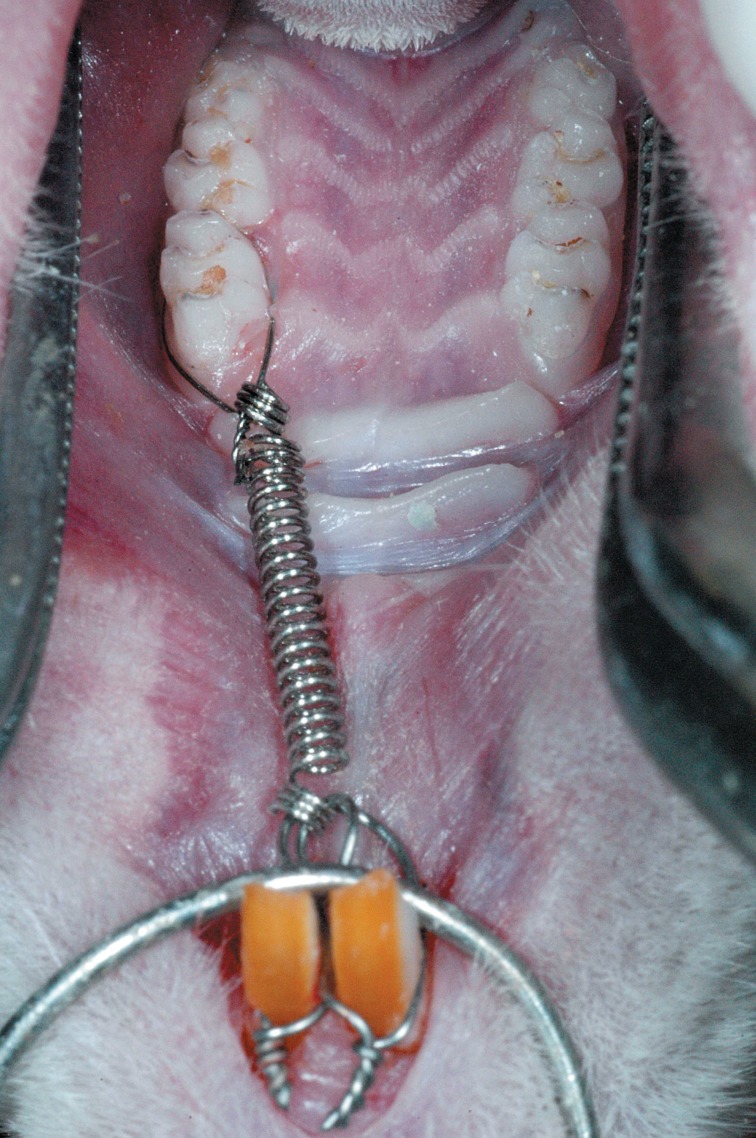
Orthodontic appliance used to achieve mesial inclination of the maxillary left first molar, anchored on the maxillary incisors, delivering a force of 75 g

Appliances were placed on the maxillary left first molars of 49 Wistar albino rats aged 120 days, divided into 7 experimental groups, according to the period of tooth movement (1 to 7 days)^13^^,^^17^. Three animals were analyzed at each period, yet nine animals were evaluated at 3, 5 and 7 days, which are the most representative periods of the biological effects of induced tooth movement. Other 10 animals were taken apart as the control group and were killed without accomplishment of any orthodontic movement. The device was placed as suggested by Heller and Nanda^13^ (1979) and improved by Martins-Ortiz^17^ (2005), and was composed of a stainless steel coil spring tied to the maxillary first molar and anchored to the maxillary incisors, which delivered 75 g of force (Figure 1). To place the device for tooth movement, the animals were anesthetized with a mixture of equal parts of ketamine hydrochloride 100 mg/mL (Dopalen – Vetbrands) and muscle relaxant xylazine hydrochloride 20 mg/mL (Anasedan – Vetbrands), at a dose of 1 mL/Kg. The solution was applied with a 1-mL syringe and 12.7-mm sterile needle by intramuscular injection.

The rats were killed by an overdose of anesthetics (ketamine and xylazine), according to the periods of tooth movement of each group. After that, the maxillae were removed with surgical scissors and the specimens were fixed in 10% buffered formalin. After fixation, the specimens were decalcified in Morse’s solution for one week, embedded in paraffin and longitudinally sectioned of 6-μm thickness. The sections were placed on glass slabs and stained with hematoxylin-eosin. Ten slabs with 5 sections each were prepared for each animal.

The mesiobuccal and distobuccal roots were analyzed on longitudinal sections. The presence of hyaline areas of periodontal ligament, frontal bone resorption and undermining bone resorption was registered for both roots of each animal. When there were extensive hyaline areas involving segments of periodontal ligament and undermining resorption, the microscopic findings were interpreted as resulting from intense force. Small hyaline areas and frontal bone resorption were interpreted as the result of biologically acceptable forces.

At the apical third, more specifically at the apical periodontal ligament, microscopic evaluation addressed the detection of circulatory changes such as blood vessel congestion, hemorrhage and thrombosis.

The main changes investigated in the dental pulp comprised cell vacuolization, tubules with nuclei, reduced cellularity, fibrosis, pulp hyalinization, pulp nodules, dystrophic calcifications, reactive dentin, blood vessel congestion, areas of hemorrhage and thrombosis. The analysis also addressed the disorganization of the odontoblast layer and areas of pulp necrosis.

### Statistical analysis

Statistical evaluation was performed between control and experimental groups and between periods of observation using non-parametric chi-square, Kruskal-Wallis and Dunn tests.

At 30 days after the first microscopic analysis, 30 specimens were randomly selected and re-evaluated; the results of the first and second analyses were compared for investigation of intra-examiner agreement by *Kappa* analysis, considering values above 0.80 as good level of agreement.

## RESULTS

Evaluation of intra-examiner agreement revealed a *kappa* value of 0.83, demonstrating the effectiveness of the analysis of microscopic sections conducted by the examiner.

Concerning the evaluation of periodontal microscopic phenomena, statistically significant differences were found in the Experimental groups compared to the Control group. This difference was found for the presence of segmental hyalinization at 3 and 5 days, at the 5% level, between control and experimental groups. The analysis of frequency of frontal bone resorption did not reveal significant differences among groups; however, undermining resorption was significantly more frequent at 5 days of induced tooth movement. Considering a lower significance level of 10%, undermining resorption was greater at 3, 5 and 7 days compared to the Control group. The most significant results were observed for the presence of root resorption at 5 and 7 days compared to the Control group (Table 1).

**Table 1:** Frequency of periodontal phenomena microscopically observed in the control and experimental groups, with induced tooth movement (ITM) on the maxillary first molars of rats

Periodontal changes	Groups							
	No ITM	1 day	2 days	3 days	4 days	5 days	6 days	7 days
	n=10	n=3	n=3	n=9	n=3	n=9	n=3	n=9
Focal hyalinization	0	2	0	2	0	2	0	3
Segmental hyalinization	0	1	3	7*	3	7*	3	6
Frontal bone resorption	0	0	1	3	1	2	0	3
Undermining resorption	0	0	0	6**	2	7*	3	6**
Root resorption	0	0	0	0	1	8*	3	9*

* Significant at the 5% level in relation to the Control group without ITM

** Significant at the 10% level in relation to the Control group without ITM

Evaluation of the Control and Experimental groups at the different periods revealed uniform morphology of the dental pulp. In both groups, the blood vessels were usually congested and filled with blood components, predominantly erythrocytes. The odontoblast layer occasionally presented cell vacuolization and loss of continuity, yet associated with artifacts. These observations on morphological normality were valid for the entire dental pulp, from the coronal to the apical region. In the internal dentinal wall, turned toward the dental pulp, there was neither thickening of the pre-dentin layer nor morphological signs of reactive dentin that could indicate any pulpal effect from the force applied throughout this experiment of induced tooth movement in rats (Table 2).

**Table 2:** Frequency of pulp phenomena microscopically observed in the control and experimental groups, with induced tooth movement (ITM) on the maxillary first molars of rats

Pulp changes	Groups							
	No ITM	1 day	2 days	3 days	4 days	5 days	6 days	7 days
	n=10	n=3	n=3	n=9	n=3	n=9	n=3	n=9
Cell vacuolization	2	0	1	2	0	0	0	0
Tubules with nuclei	0	0	0	0	0	0	0	0
Reduced cellularity	0	0	0	0	0	0	0	0
Increased fibrosis	0	0	0	0	0	0	0	0
Hyalinization	0	0	0	0	0	0	0	0
Pulp nodules	1	0	0	0	0	0	0	0
Diffuse calcification	0	0	0	0	0	0	0	0
Reactive dentin	0	0	0	0	0	0	0	0
Blood vessel congestion	10	3	2	8	3	9	2	9
Hemorrhage	0	0	0	0	0	0	0	0
Thrombosis	0	0	0	0	0	0	0	0

* Significant at the 5% level in relation to the Control group without ITM

** Significant at the 10% level in relation to the Control group without ITM

## DISCUSSION

**Figure 2:**
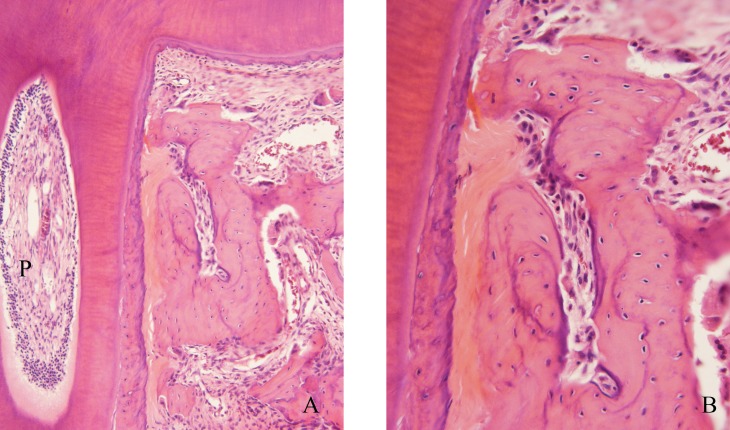
Periodontal morphological changes induced on the cervical region of the distobuccal root submitted to intense forces (A and B). Experimental group, after 3 days of induced tooth movement on the maxillary first molar of rats. (A) Note the normal aspect of the pulp (P). A: 25 x magnification; B: 40 x magnification

**Figure 3:**
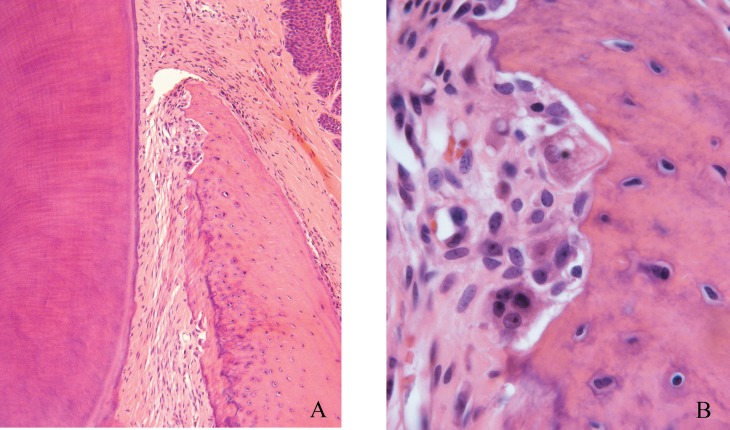
Periodontal morphological changes induced on the cervical region of the mesiobuccal root (A and B) submitted to moderate forces with frontal bone resorption. Experimental group, after 3 days of induced tooth movement on the maxillary first molar of rats. A: 25 x magnification; B: 40 x magnification

The forces applied to the maxillary first molars of rats were intense on the mesial interseptal surface of the distobuccal root (Figure 2) and moderate on the mesial region of the mesiobuccal root, both analyzed on the cervical third (Figure 3).

Analysis of the distobuccal root microscopically demonstrated the biological effects of application of intense forces, with presence of hyaline areas of periodontal ligament at 3 to 5 days of induced tooth movement, especially on the cervical region at the pressure side (Figure 2 and 5). At 5 days and especially at 7 days, on the same region, there was higher frequency of root resorption (Figure 5). The forces applied to the mesiobuccal root had moderate intensity, with absence of hyalinization of the periodontal ligament and root resorption at the most advanced periods of induced tooth movement (Figure 3).

**Figure 4:**
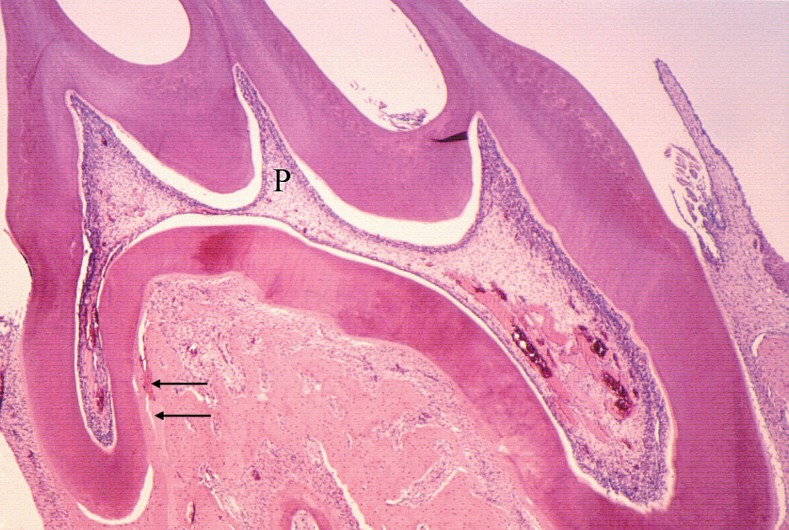
Microscopic aspects of the pulp and periodontal morphological changes in the periodontal ligament, after 4 days of induced tooth movement on the maxillary first molar of rats. Note the segmental hyalinization of periodontal ligament on the distal root (arrows) and the normal aspect of the pulp (P). Pulp vascular congestion was due to operatory procedures during material collecting and histotechnical process. 10 x magnification

**Figure 5:**
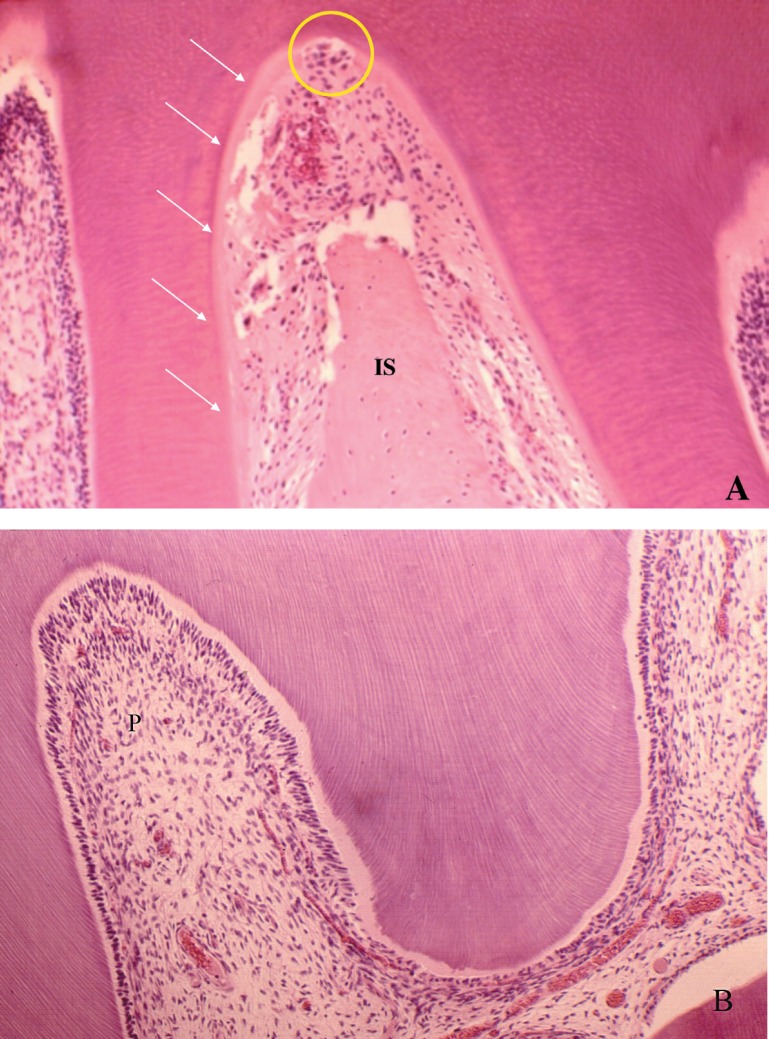
Microscopic aspects of the root pulp (P) in A, and coronal pulp in B, compatible with normal morphology of maxillary first molar of rat submitted to induced tooth movement for 7 days. On the interseptal surface (IS) of the distobuccal root, the cementoblast layer was extensively lost (arrows), with presence of several clasts and areas of root resorption (circle). A: 25 x magnification; B: 25 x magnification

The results revealed that the forces generated during experimental induced tooth movement were not able to cause changes in the pulp tissue even if intense, as observed in the initial periods up to 7 days (Table 2, Figure 2, 4 and 5).

The experimental model of induced tooth movement adequately reproduces the biological situation found in humans. However, the metabolic rate of connective tissues of rats should be considered when using this experimental model. A period of 21 days is required for the observation of all stages of induced tooth movement in humans, whereas in rats this period is reduced to 7 days^11^.

Previous studies in the literature^1^^,^^4^^,^^19^^,^^25^^,^^27^^,^^28^ indicate that orthodontic movement does not induce pulp changes such as increased number of pulp modules, calcific metamorphosis of the pulp, permanent vascular changes, or early pulp aging represented by hyalinization of the extracellular matrix and fibrosis. These changes are generically called regressive and/ or degenerative^8^^,^^24^.

Investigations reveal the occurrence of several biological reactions during induced tooth movement, including increased^15^ or reduced^14^^,^^18^ blood flow; reduced^12^^,^^31^ or increased^16^ cell respiration; and increased angiogenesis^9^^,^^10^. However, changes in the local level of enzymes and mediators observed in cultures and laboratory biochemical tests on the pulp tissue^23^^,^^32^ indicate possible changes in the metabolic rates of the pulp tissue, rather than loss of biological viability or vitality. These metabolic changes did not morphologically changes the pulp tissue.

Clinical cases^8^^,^^30^ should properly include all records, from photographs to periapical radiographs, before and during orthodontic treatment, or rule out the possibility of dental trauma^5^^,^^6^^,^^19^.

For the endodontist and orthodontist, the pulp status should be precisely and safely determined before the onset of orthodontic treatment. After diagnosis of pulp status of teeth which will be submitted to orthodontic movement, treatment may be performed in a safe manner and with good prognosis. In teeth without caries, restorations or periodontal disease, trauma should be investigated as the main suspected cause of pulp changes during and after orthodontic treatment.

Pulp necrosis induced by trauma may be diagnosed during orthodontic treatment. The tissue changes inherent to tooth movement may exacerbate the clinical and radiographic signs of chronic pericementitis and periapical granuloma, not diagnosed on the initial orthodontic records. The initial correct diagnosis of the teeth should be done with periapical radiographic.

When compared to traumatisms or mechanic forces of different kinds, orthodontic forces can be considered little or small, even when called heavy or intense^5^^,^^6^. Studies comprising experimental intrusion and tooth movement with forces considered intense did not produce pulp necrosis in a similar context as the orthodontic treatment^4^^,^^25^.

In the present study, the effectiveness of the force applied was demonstrated by the microscopically observable changes in the periodontal tissue, including significant loss of segmental hyalinization at 3 to 5 days, undermining resorption at 5 days and root resorption at 7 days; however, the pulps of moved teeth did not present any degenerative or inflammatory change (Figures 2, 4 and 5). The odontoblast layer occasionally presented cell vacuolization and loss of continuity, yet associated to artifacts.

Therefore, according to the present results and previous reports in the literature, it is suggested that clinical cases of pulp necrosis occurring during and after orthodontic treatment should be managed with greater emphasis on the dental history of the patient and possibility of trauma, rather than on the orthodontic force applied.

## CONCLUSION

Based on the present results and considering the inherent limitations of this experimental model, it may be concluded that induced tooth movement does not promote microscopically observable degenerative or inflammatory morphological changes in the dental pulp.

## References

[1] Anstendig HS, Kronman JH (1972). A histologic study of pulpal reaction to orthodontic tooth movement in dogs. Angle Orthod.

[2] Barwick PJ, Ramsay DS (1996). Effect of brief intrusive force on human pulpal blood flow. Am J Orthod Dentofac Orthop.

[3] Butcher EO, Taylor C (1951). The effects of denervation and ischemia upon the teeth of the monkey. J Dent Res.

[4] Butcher EO, Taylor C (1952). The vascularity of the incisor pulp of the monkey and its alteration by tooth retraction. J Dent Res.

[5] Consolaro A (2002). Movimento ortodôntico não promove necrose pulpar. R Clin Ortodon Dental Press.

[6] Consolaro A (2005). Reabsorções dentárias nas especialidades clínicas.

[7] Davidovitch Z (1991). Tooth movement. Crit Rev Oral Biol Med.

[8] Delivanis HP, Sauer GJ (1982). Incidence of canal calcification in the orthodontic patient. Am J Orthod Dentofac Orthop.

[9] Derringer KA, Jaggers DC, Linden RWA (1996). Angiogenesis in human dental pulp following orthodontic tooth movement. J Dent Res.

[10] Derringer KA, Liden RW (1998). Enhance angiogenesis growth induced by diffusible angiogenic growth factors release from human dental pulp explants of orthodontically moved teeth. Eur J Orthod.

[11] Farris H, Griffith R, Farris H, Griffith R (1963). The teeth. The rat in laboratory investigation.

[12] Hamersky PA, Weimer AD, Taintor JF (1980). The effect of orthodontic force application on the pulpal tissue respiration rate in the human premolar. Am J Orthod Dentofac Orthop.

[13] Heller IJ, Nanda R (1979). Effect of metabolic of periodontal fibers on orthodontic tooth movement. Am J Orthod.

[14] Ikawa M, Fujiwara M, Horiuchi H, Shimauchi H (2001). The effect of short-term tooth intrusion on human pulpal blood flow measured by laser Doppler flowmetry. Arch Oral Biol.

[15] Kvinnsland S, Heyeraas K, Ofjord ES (1989). Effect of experimental tooth movement on periodontal and pulpal blood flow. Eur J Orthod.

[16] Labart WA, Taintor JF, Dyer JK, Weimer AD (1980). The effect of orthodontic forces on pulp respiration in the rat incisor. J Endod.

[17] Martins-Ortiz MF, Consolaro A, Consolaro A (2005). Influência dos bisfosfonatos na movimentação dentária induzida e nas reabsorções dentárias associadas. Reabsorções dentárias nas especialidades clínicas.

[18] McDonald F, Pitt Ford TR (1994). Blood flow changes in permanent maxillary canines during retraction. Eur J Orthod.

[19] Mjör IA, Stenvik A (1969). Microradiography and histology of decalcified human teeth following experimental intrusion; with emphasis on resorption. Arch Oral Biol.

[20] Oppenheim A (1936). Biologic orthodontic therapy and reality. Angle Orthod.

[21] Oppenheim A (1937). Biologic orthodontic therapy and reality. Angle Orthod.

[22] Oppenheim A (1942). Human tissue response to orthodontic intervention of short and long duration. Am J Orthod.

[23] Perinetti G, Varvara G, Festa F, Esposito P (2004). Aspartate aminotransferase activity in pulp of orthodontic tooth movement in adolescents: a radiographic study. Am J Orthod Dentofac Orthop.

[24] Popp TW, Artun J, Linge L (1992). Pulpal response to orthodontic tooth movement in adolescents: a radiographic study. Am J Orthod Dentofac Orthop.

[25] Ramazanzadeh BA, Sahhafian AA, Mohtasham N, Hassanzadeh N, Jahanbin A, Shakeri MT (2009). Histological changes in human dental pulp following application of intrusive and extrusive orthodontic forces. J Oral Sci.

[26] Sano Y, Ikawa M, Sugawara J, Horiuchi H, Mitani H (2002). The effect of continuous intrusive force on human pulpal blood flow. Eur J Orthod.

[27] Santamaria M, Milagres D, Iyomasa MM, Stuani MBS, Ruellas ACO (2007). Initial pulp changes during orthodontic movement: histomorphological evaluation. Braz Dent J.

[28] Santamaria M, Milagres D, Stuani AS, Stuani MBS, Ruellas ACO (2006). Pulpal vasculature changes in tooth movement. Eur J Orthod.

[29] Strang RHW (1943). A textbook of orthodontia.

[30] Stuteville OH (1938). Injuries caused by orthodontic forces and the ultimate results of these injuries. Am J Orthod Oral Surg.

[31] Unterseher RE, Neiberg LG, Weimer AD, Dyer JK (1987). The response of human pulp tissue after orthodontic force application. Am J Orthod Dentofac Orthop.

[32] Walker JA, Tanzer FS, Harris EF, Wakelyn C, Desiderio DM (1987). The enkephalin response in human tooth pulp to orthodontic force. Am J Orthod Dentofac Orthop.

